# Supplementary figures and images for: p75NTR prevents the onset of cerebellar granule cell migration via RhoA activation
Source: eLife. 2022 Aug 30;11:e79934. doi: 10.7554/eLife.79934 (PMC9427104; doi:10.7554/eLife.79934)

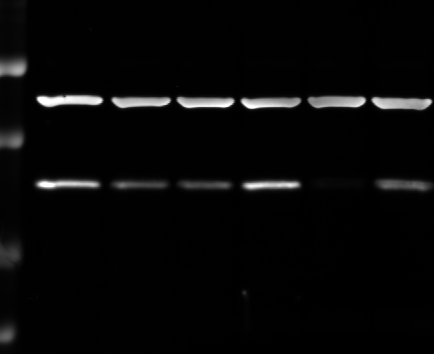

Supplement: Figure 2—source data 1. [file elife-79934-fig2-data1.zip › Figure 2 - Source Data 1/RAW Actin.tif]

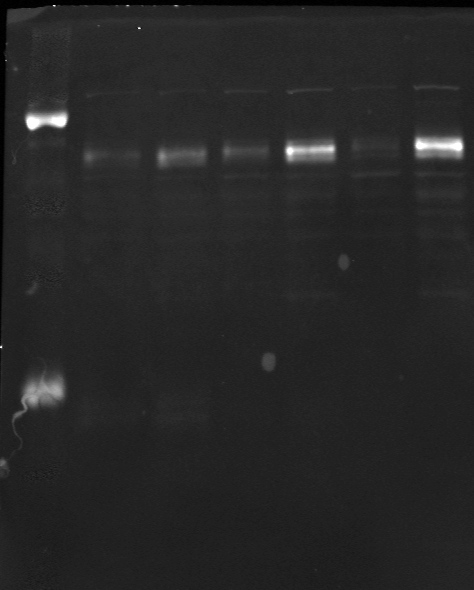

Supplement: Figure 2—source data 1. [file elife-79934-fig2-data1.zip › Figure 2 - Source Data 1/RAW P75.tif]

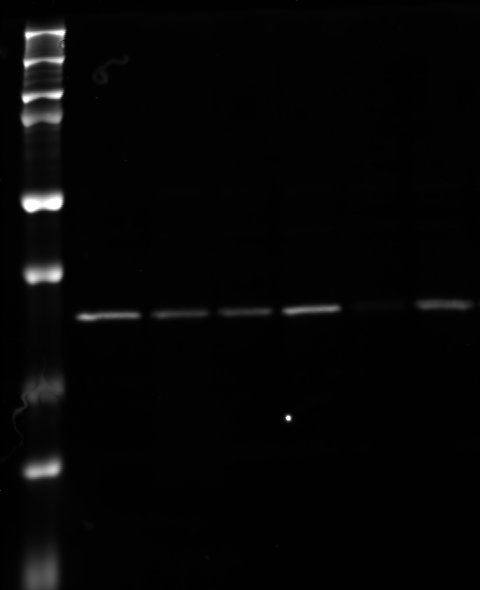

Supplement: Figure 2—source data 1. [file elife-79934-fig2-data1.zip › Figure 2 - Source Data 1/RAW PCNA.tif]

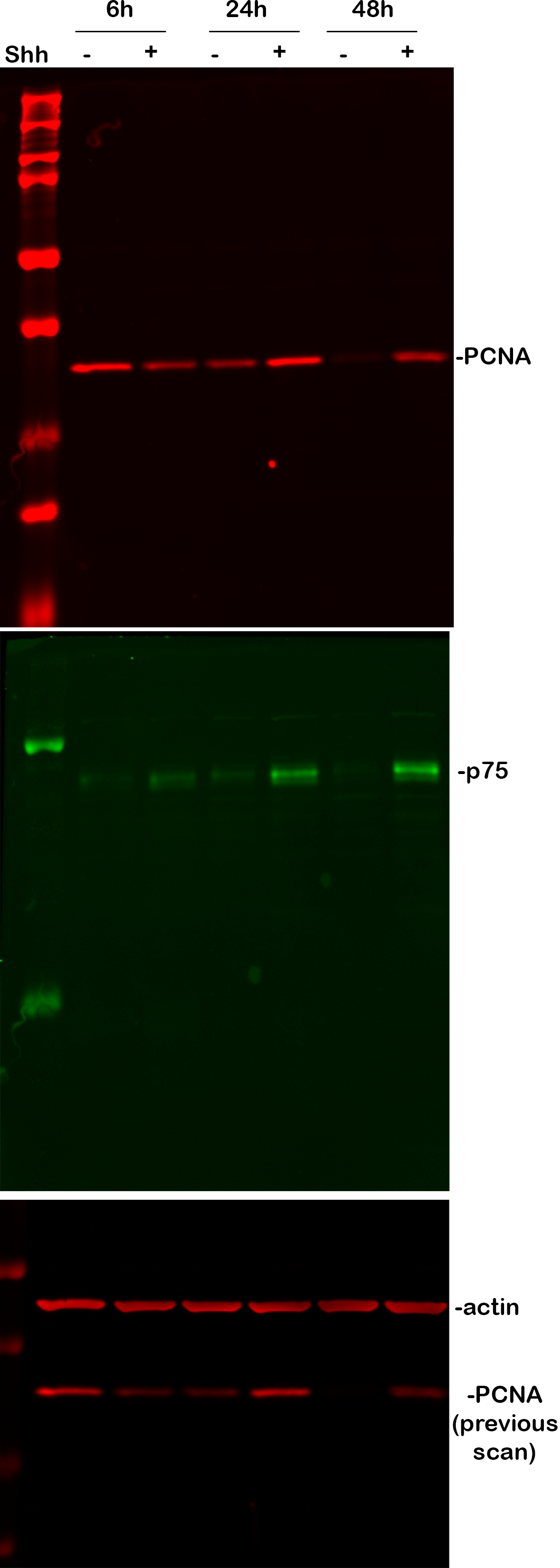

Supplement: Figure 2—source data 1. [file elife-79934-fig2-data1.zip › Figure 2 - Source Data 1/WB Figure 2 Shh temporal.tif]

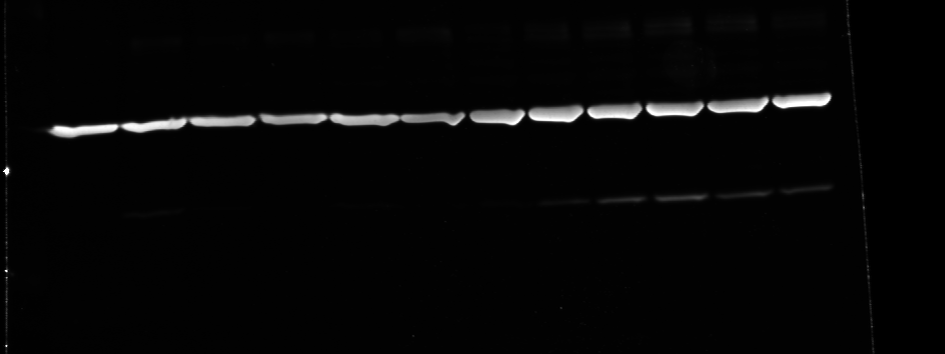

Supplement: Figure 2—source data 3. [file elife-79934-fig2-data3.zip › Figure 2 - Source Data 3/RAW Actin.TIF]

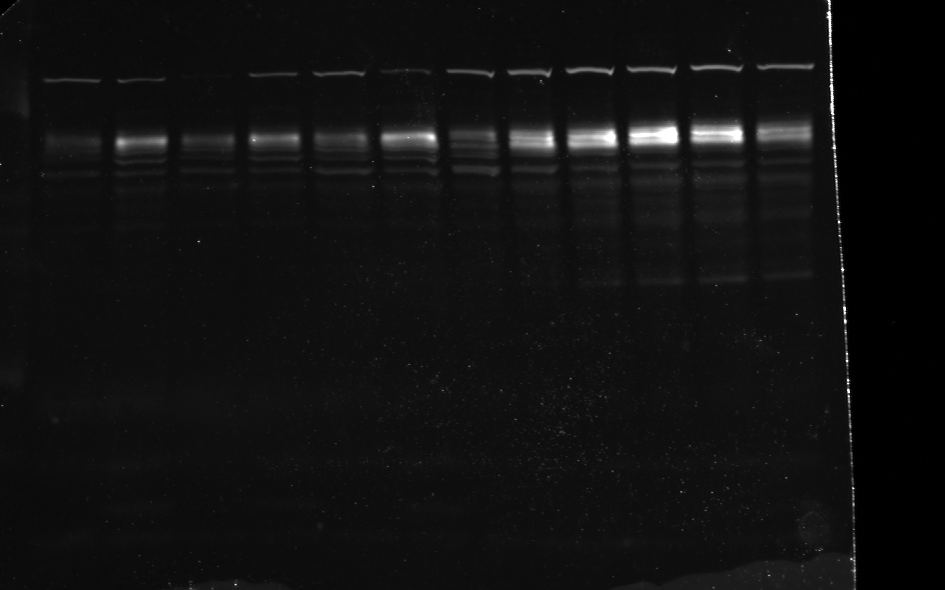

Supplement: Figure 2—source data 3. [file elife-79934-fig2-data3.zip › Figure 2 - Source Data 3/RAW p75.TIF]

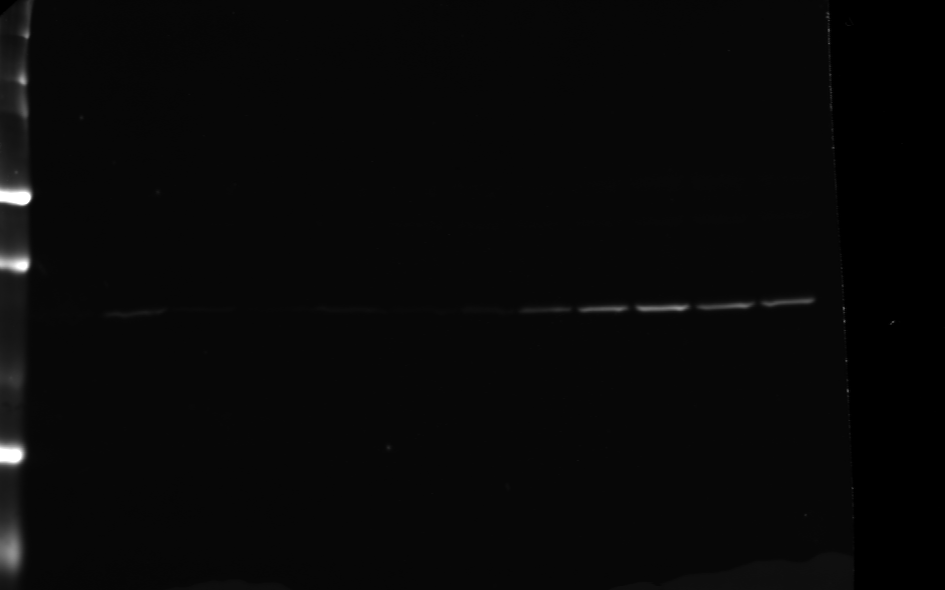

Supplement: Figure 2—source data 3. [file elife-79934-fig2-data3.zip › Figure 2 - Source Data 3/RAW PCNA.TIF]

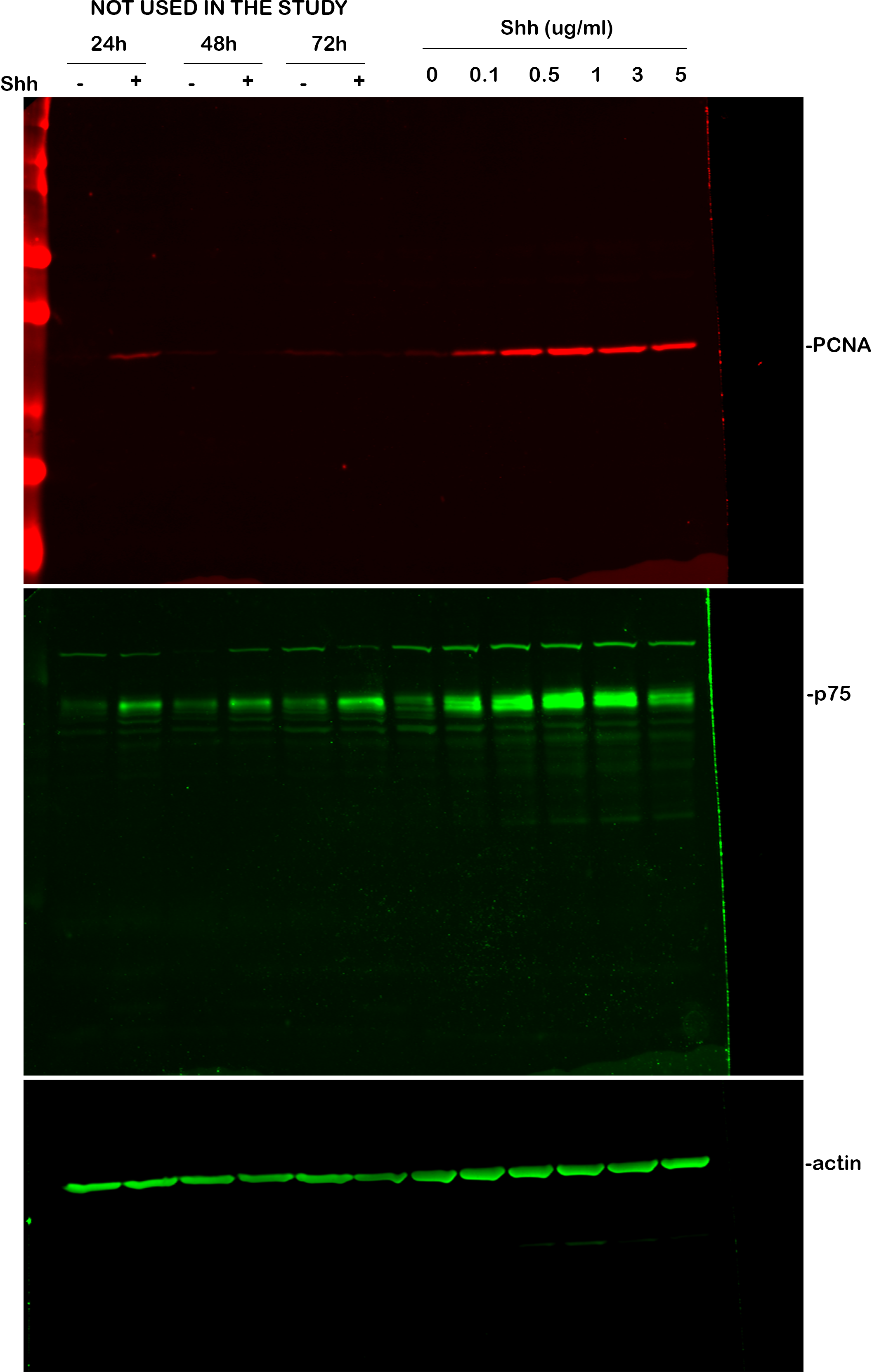

Supplement: Figure 2—source data 3. [file elife-79934-fig2-data3.zip › Figure 2 - Source Data 3/WB Figure 2 Shh Dose response.tif]

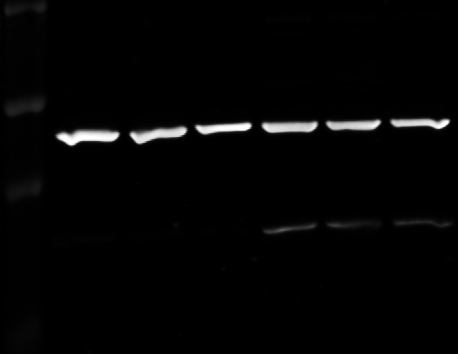

Supplement: Figure 3—source data 3. [file elife-79934-fig3-data3.zip › Figure 3 - Source Data 3/RAW Actin.tif]

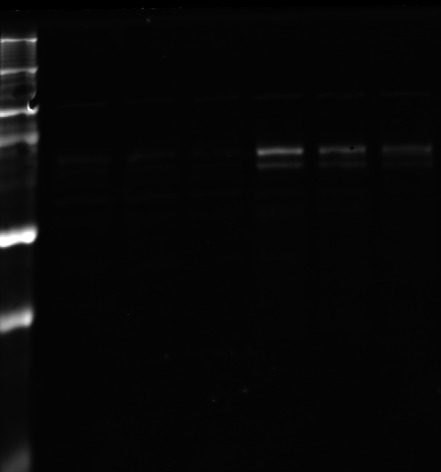

Supplement: Figure 3—source data 3. [file elife-79934-fig3-data3.zip › Figure 3 - Source Data 3/RAW p75.tif]

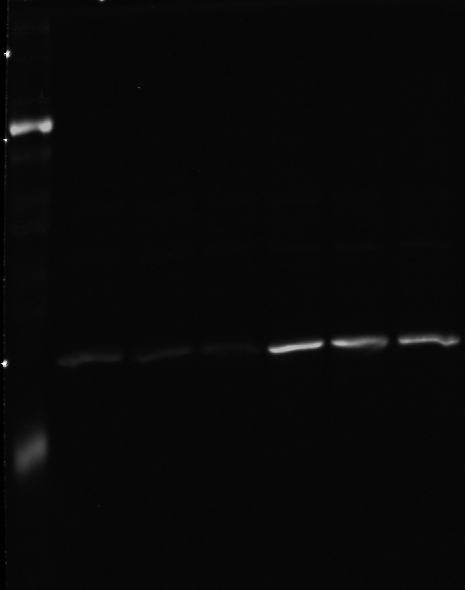

Supplement: Figure 3—source data 3. [file elife-79934-fig3-data3.zip › Figure 3 - Source Data 3/RAW PCNA.tif]

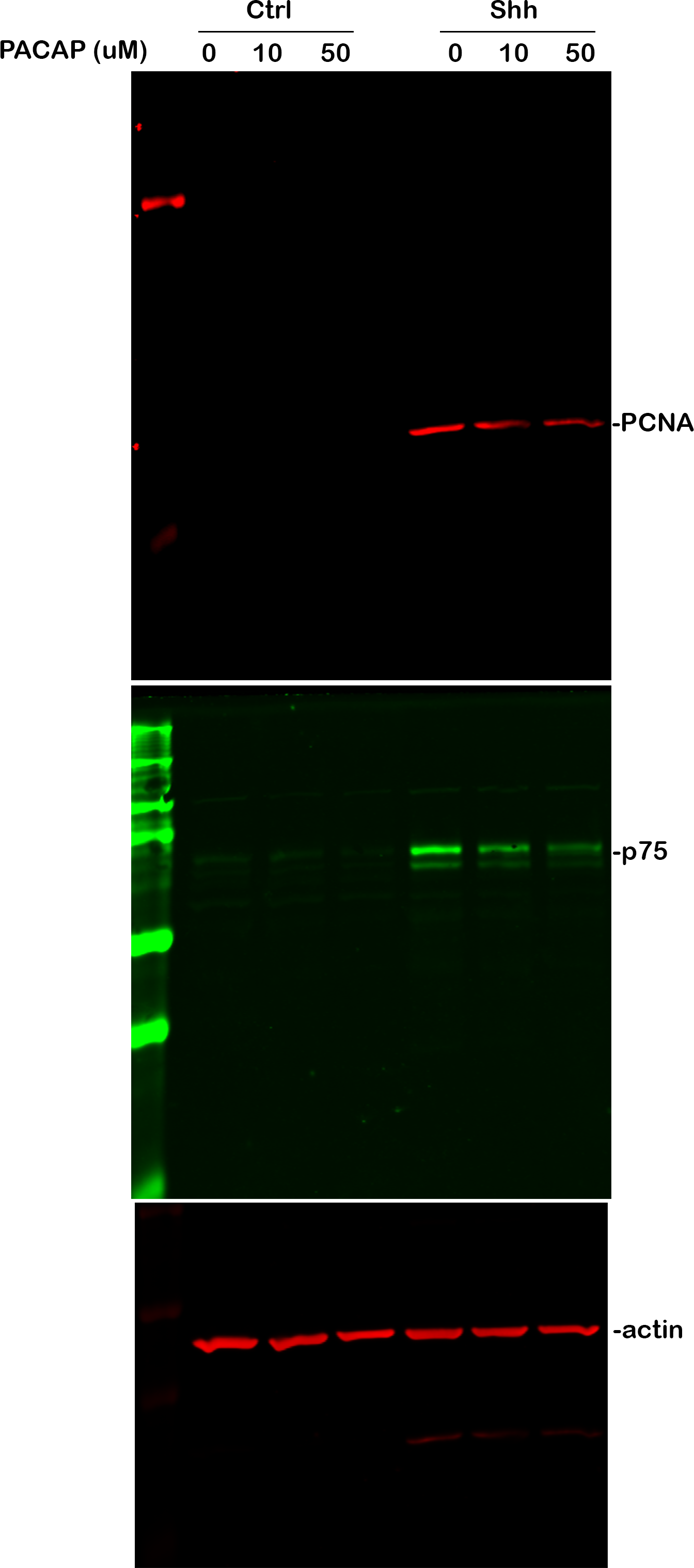

Supplement: Figure 3—source data 3. [file elife-79934-fig3-data3.zip › Figure 3 - Source Data 3/WB Figure 3 Shh PACAP.tif]

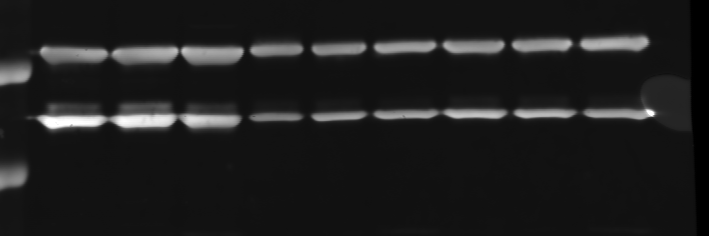

Supplement: Figure 4—source data 2. [file elife-79934-fig4-data2.zip › Figure 4 - Source Data 2/RAW Actin.TIF]

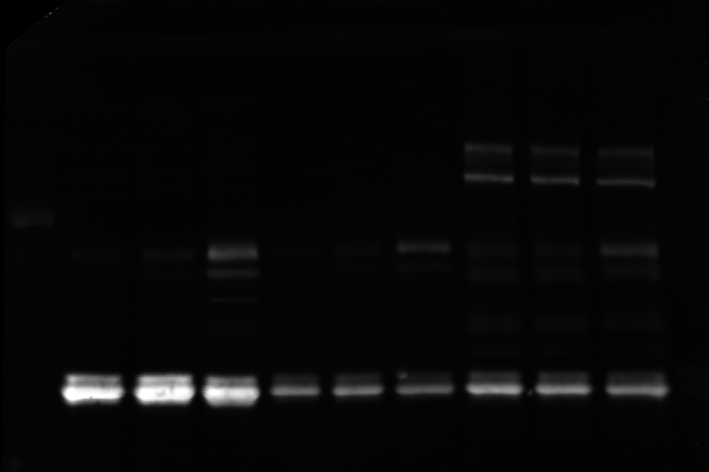

Supplement: Figure 4—source data 2. [file elife-79934-fig4-data2.zip › Figure 4 - Source Data 2/RAW p75.TIF]

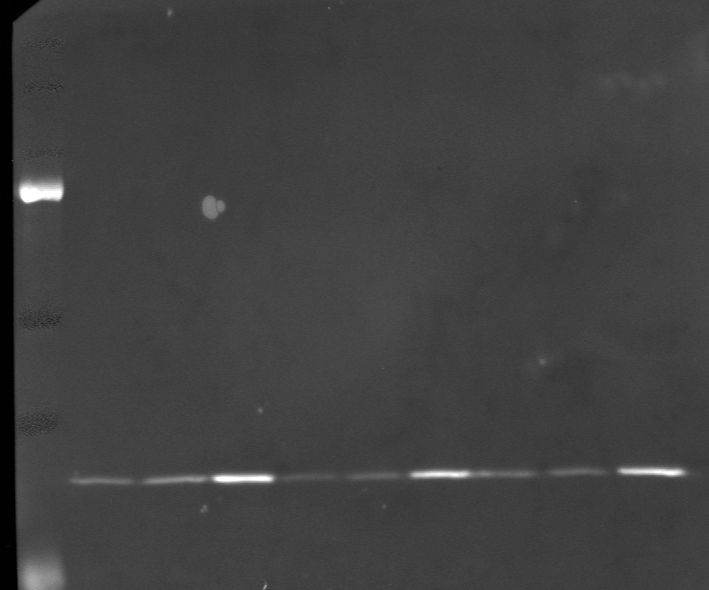

Supplement: Figure 4—source data 2. [file elife-79934-fig4-data2.zip › Figure 4 - Source Data 2/RAW PCNA.TIF]

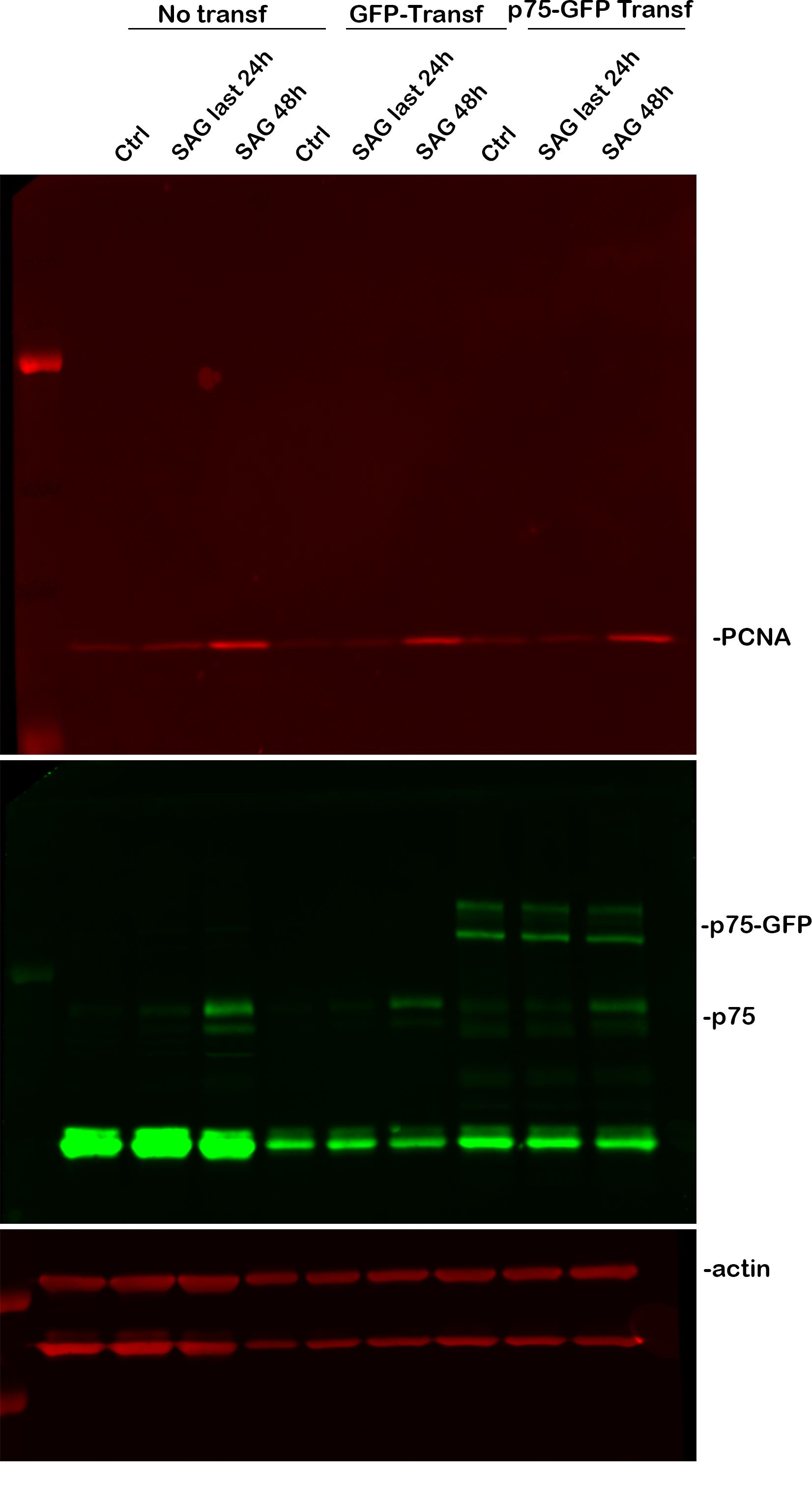

Supplement: Figure 4—source data 2. [file elife-79934-fig4-data2.zip › Figure 4 - Source Data 2/WB Figure 4 p75 Transfection.tif]
